# Dihydroxy‐Acid Dehydratases From Pathogenic Bacteria: Emerging Drug Targets to Combat Antibiotic Resistance

**DOI:** 10.1002/chem.202200927

**Published:** 2022-06-16

**Authors:** Tenuun Bayaraa, Jose Gaete, Samuel Sutiono, Julia Kurz, Thierry Lonhienne, Jeffrey R. Harmer, Paul V. Bernhardt, Volker Sieber, Luke Guddat, Gerhard Schenk

**Affiliations:** ^1^ School of Chemistry and Molecular Biosciences The University of Queensland Brisbane 4072 Australia; ^2^ Chair of Chemistry of Biogenic resources Campus Straubing for Biotechnology and Sustainability Technical University of Munich Schulgasse 16 94315 Straubing Germany; ^3^ Centre for Advanced Imaging The University of Queensland Brisbane 4072 Australia; ^4^ Sustainable Minerals Institute The University of Queensland Brisbane 4072 Australia; ^5^ Australian Institute for Bioengineering and Nanotechnology The University of Queensland Brisbane 4072 Australia

**Keywords:** antibiotics, dihydroxy-acid dehydratase, enzyme activation, Fe−S cluster, sustainable chemistry

## Abstract

There is an urgent global need for the development of novel therapeutics to combat the rise of various antibiotic‐resistant superbugs. Enzymes of the branched‐chain amino acid (BCAA) biosynthesis pathway are an attractive target for novel anti‐microbial drug development. Dihydroxy‐acid dehydratase (DHAD) is the third enzyme in the BCAA biosynthesis pathway. It relies on an Fe−S cluster for catalytic activity and has recently also gained attention as a catalyst in cell‐free enzyme cascades. Two types of Fe−S clusters have been identified in DHADs, i.e. [2Fe−2S] and [4Fe−4S], with the latter being more prone to degradation in the presence of oxygen. Here, we characterise two DHADs from bacterial human pathogens, *Staphylococcus aureus* and *Campylobacter jejuni* (*Sa*DHAD and *Cj*DHAD). Purified *Sa*DHAD and *Cj*DHAD are virtually inactive, but activity could be reversibly reconstituted in vitro (up to ∼19,000‐fold increase with *k_cat_* as high as ∼6.7 s^−1^). Inductively‐coupled plasma‐optical emission spectroscopy (ICP‐OES) measurements are consistent with the presence of [4Fe−4S] clusters in both enzymes. N‐isopropyloxalyl hydroxamate (IpOHA) and aspterric acid are both potent inhibitors for both *Sa*DHAD (*K*_i_=7.8 and 51.6 μM, respectively) and *Cj*DHAD (*K*_i_=32.9 and 35.1 μM, respectively). These compounds thus present suitable starting points for the development of novel anti‐microbial chemotherapeutics.

## Graphical abstract

The FeS cluster‐dependent dihydroxy‐acid dehydratases (DHADs) from *Staphylococcus aureus* and *Campylobacter jejuni* (*Sa*DHAD and *Cj*DHAD) are emerging targets for biocides. They are virtually inactive after extraction, but a functional [4Fe−4S] cluster can be reconstituted in vitro boosting the activity up to ∼19,000‐fold.

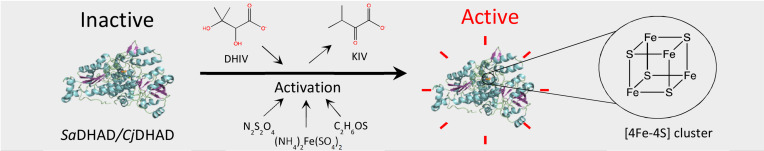

## Introduction

**Figure 1:**
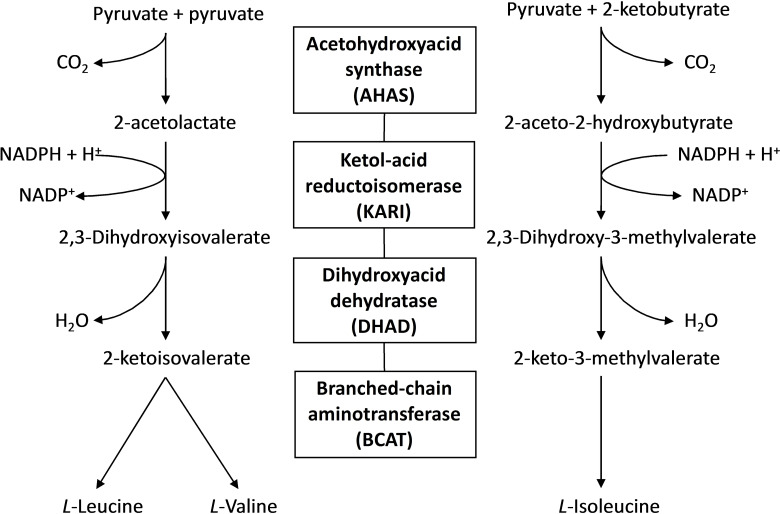
Overview of the branched chain amino acid (BCAA) biosynthesis pathway.

The rapid increase in antibiotic resistance among human pathogens has become a major global threat. It is well established that new lines of drugs are urgently needed to combat resistance. The branched chain amino acids (BCAAs), valine, leucine and isoleucine are synthesized de novo through the BCAA pathway (Figure 1) in bacteria, plant and fungi.[^1^, ^2^] The BCAAs are crucial building blocks of nearly all proteins and are thus necessary for the survival of all organisms. However, this pathway is not present in animals, including humans, which obtain BCAAs directly from their diet.[^1^, ^2^] Therefore, the enzymes of the BCAA biosynthesis pathway are excellent targets for antimicrobial drug discovery and herbicide development since their absence in the animal kingdom is anticipated to minimize side effects caused by their inhibition by biocides.[^1^, ^2^, ^3^, ^4^] The first three enzymes of this pathway are acetohydroxyacid synthase (AHAS)^[5]^ ketol‐acid reductoisomerase (KARI)[^6^, ^7^, ^8^, ^9^] and dihydroxyacid dehydratase (DHAD).[^10^, ^11^, ^12^, ^13^]

AHAS has been used effectively as a target of more than 50 commercial herbicides with some of the inhibitors also showing antimicrobial activity.[^3^, ^4^] Importantly, sulfometuron methyl, a commercial AHAS herbicide, has been shown to have anti‐tuberculosis (TB) activity.^[14]^ Other novel AHAS inhibitors have also been shown to have anti‐TB activity within the range 0.38 to 200 μM in in vitro assays.^[15]^ More recently, KARI has also emerged as a promising drug target;[^6^, ^8^, ^16^, ^17^] in both *Mycobacterium tuberculosis* (*Mt*) and uropathogenic *Escherichia coli* (*Ec*), KARI has been shown to be essential for growth and survival.[^16^, ^17^] Consequently, diverse compounds that significantly inhibit KARI have recently been developed as potential anti‐microbial drug candidates.[^8^, ^18^, ^19^, ^20^, ^21^, ^22^]

DHAD, the third enzyme in the BCAA biosynthesis pathway, has received less attention than AHAS and KARI. It belongs to the ilvD/EDD superfamily that also includes sugar acid dehydratases (DHTs).[^12^, ^13^, ^23^, ^24^] One of the main characteristics of this family is the presence of an Fe−S cluster in the active site, that is essential for the catalytic function of these enzymes.^[23]^ Two types of Fe−S clusters have been observed, i.e. [2Fe−2S] and [4Fe−4S].[^10^, ^11^, ^25^] The [4Fe−4S] cluster was first observed in DHAD from *E. coli*,^[11]^ while the [2Fe−2S] cluster was first reported for spinach DHAD.^[10]^ The [4Fe−4S] cluster is sensitive to oxygen, with oxidation leading to enzyme inactivation.^[11]^ The [2Fe−2S] cluster, on the other hand, is more stable in the presence of oxygen.[^10^, ^23^, ^24^, ^26^]

**Figure 2:**
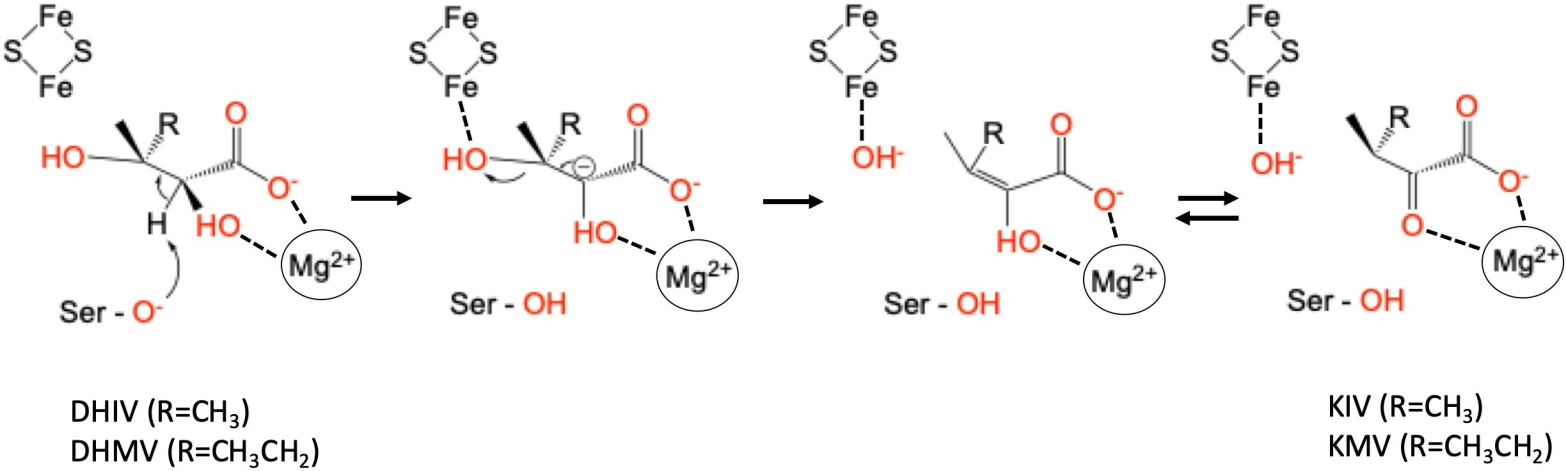
Proposed mechanism of a DHAD with a [2Fe−2S] cluster. The Fe−S cluster acts as a Lewis acid, while an active site residue (a conserved deprotonated serine) acts as a Lewis base to produce KIV (R=CH_3_) and KMV (R=CH_3_CH_2_).

DHAD catalyses the dehydration of dihydroxy‐isovalerate (DHIV) or dihydroxy‐methylvalerate (DHMV) to keto‐isovalerate (KIV) and keto‐methylvalerate (KMV), respectively (Figure 2).[^25^, ^27^] It is proposed that the dehydration reaction is initiated by the abstraction of the proton at the C2 position of the substrate by the alkoxide side chain of a conserved serine residue in the active site. The resulting carbanion may be stabilized by the Mg^2+^ ion in the vicinity of the Fe−S cluster. This Mg^2+^ ion is also essential for the catalytic activity of DHADs.[^11^, ^25^, ^28^] The combined effect of this proton abstraction and the coordination of the alcohol moiety at position C3 of the substrate to one of the iron atoms of the Fe−S cluster leads to a weakening of the C3−OH bond and its subsequent cleavage (Figure 2). A tautomerization completes the catalytic reaction, leading to the keto product.

**Figure 3:**
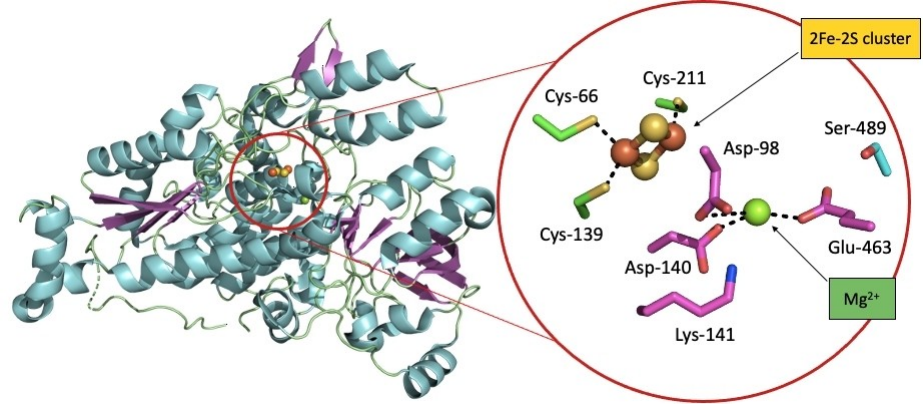
Crystal structure of a DHAD monomer from *A. thaliana*.^[29]^ The active site is zoomed in on the right (red circle). The Fe−S cluster is shown in orange and yellow spheres. The Mg^2+^ ion is shown as a green sphere. The Fe−S cluster‐coordinating cysteine residues are shown as green sticks. The Mg^2+^‐coordinating residues are shown as magenta sticks. The catalytically essential serine residue is shown in cyan sticks.

The crystal structures of DHADs from *Arabidopsis thaliana* (*At*) (Figure 3) and *M. tuberculosis* were recently reported; both contain a [2Fe−2S] cluster in their active sites.[^25^, ^28^, ^29^] In both structures, the residues coordinating the [2Fe−2S] cluster and Mg^2+^ ion are fully conserved.[^28^, ^29^] A similar active site geometry has also been observed for the two DHTs, i.e. the D‐xylonate DHT from *Caulobacter crescentus* and the L‐arabinonate DHT from *Rhizobium leguminosarum*.[^23^, ^24^] However, to date no crystal structure of a DHAD or DHT with a [4Fe−4S] cluster has been reported.

DHAD and DHT have been shown to play a central role in cell‐free enzyme cascades that facilitate the conversion of glucose to high‐value products such as isobutanol.[^12^, ^13^, ^30^, ^31^, ^32^] Recent studies have also highlighted DHAD to be an essential enzyme for the growth and survival of microbes and plants; its inhibition by the natural product aspterric acid (*K*_i_=9 nM) greatly impairs the growth of harmful cyanobacteria.^[26]^ Aspterric acid is also a potent inhibitor of both *At*DHAD (*K*_i_=0.3 μM) and *Mt*DHAD (*K*_i_=10.1 μM) DHAD.[^28^, ^29^] Furthermore, the deletion of the gene encoding DHAD in *Aspergillus fumigatus* was shown to strongly reduce the virulence of this fungal pathogen.^[33]^

Here, the DHADs from the two bacterial pathogens, *Staphylococcus aureus* and *Campylobacter jejuni* (*Sa*DHAD and *Cj*DHAD), were recombinantly expressed, purified and their catalytic properties investigated and compared to those of other DHADs. *S. aureus* is responsible for one of the most common causes of bacterial infections around the world. It can cause numerous medical conditions that range from minor skin conditions to necrotizing fasciitis, sepsis and even death.[^34^, ^35^, ^36^] In particular, the recent emergence of methicillin‐resistant *S. aureus* (MRSA) has caused significant problems in the health care sector.^[36]^ *C. jejuni* is a food‐borne pathogen that causes gastrointestinal infections and can lead to Guillan‐Barre syndrome.[^37^, ^38^, ^39^] The haphazard use of antibiotics fed to farm animals has led to a rise of quinolone‐ and macrolide‐resistant *C. jejuni*, diminishing the potency and number of available antibiotics.[^37^, ^39^] Therefore, there is an urgent need to develop novel antimicrobials to combat the rise of antibiotic‐resistant *S. aureus* and *C. jejuni* as well as other emerging superbugs.

## Results and Discussion

### Expression and purification

*E. coli* BL21 strains were transformed with pET28a vectors (Figure S1) containing the genes that encode DHADs from *S. aureus* or *C. jejuni* (*Sa*DHAD or *Cj*DHAD, respectively). The DNA and protein sequences of *Sa*DHAD and *Cj*DHAD are also included in the Supporting Information. Successful transformants were selected based on their resistance to kanamycin. The recombinant expression (Figure S2) of the target enzymes was performed in autoinduction media, and the proteins were purified as described in the experimental section (Figures S3 and S4), resulting in a yield of approximately 50 mg/L for both *Sa*DHAD and *Cj*DHAD. The calculated molecular weights of *Sa*DHAD and *Cj*DHAD are 60.021 kDa and 60.104 kDa, respectively, in good agreement with SDS PAGE analyses (Figures S3 and S4). Protein samples were concentrated to ∼20 mg/mL and frozen at −80 °C in the presence of 10 % glycerol.

### Characterisation of *Sa*DHAD and *Cj*DHAD as initially purified

**Figure 4:**
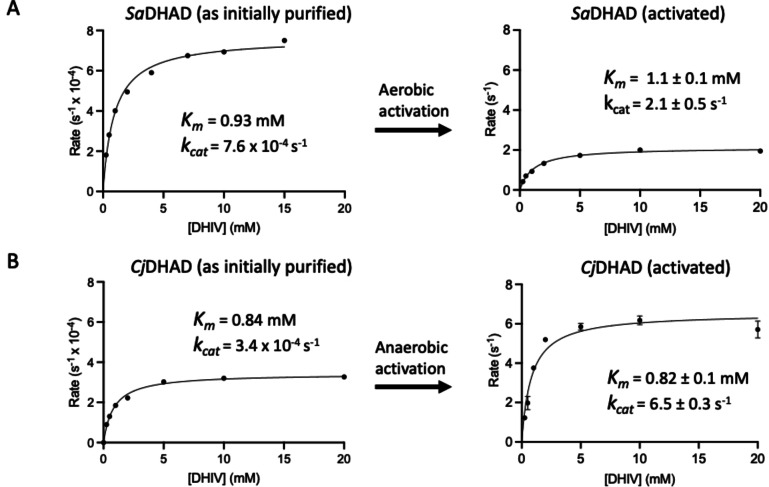
Michaelis‐Menten plots of the reaction rates of *Sa*DHAD (A) and *Cj*DHAD (B). Catalytic parameters (*K*_M_ and *k*_cat_) were measured using the natural substrate, DHIV, for both the enzymes as initially purified and their activated counterparts. Note that the catalytic activities (shown on the y‐axes) of the initially purified and activated enzymes differ by four orders of magnitude.

The reported catalytic rates (*k*_cat_ values) for the reaction of DHADs with their natural substrate, DHIV (Figure 2), vary from values as low as 0.3 s^−1^ for DHAD from *Sulfolobus solfataricus* (*Ss*DHAD) to 70 s^−1^ for the DHAD from *E. coli* (*Ec*DHAD).[^11^, ^30^] By comparison, the measured catalytic rates of *Sa*DHAD and *Cj*DHAD indicate that both enzymes are very slow, with *k*_cat_ values of 7.6×10^−4^ s^−1^ and 3.5×10^−4^ s^−1^, respectively (Figure 4). It should, however, be pointed out that previous studies with *Ss*DHAD demonstrated that the enzyme activity may be reversibly lost during the purification procedure.^[30]^ Subsequent incubation of that enzyme with the reducing agent, 2‐mercaptoethanol (2‐ME), led to a ∼threefold increase in the activity of that enzyme.^[30]^ However, further activation attempts in the presence of added Fe^2+^ led to a 78 % loss of enzymatic activity, and it was proposed that storage and purification leads to a gradual disintegration/dissociation of the Fe−S cluster from the active site.^[30]^ The difficulty in obtaining and maintaining a fully active DHAD was further illustrated by a recent study with a [2Fe−2S] cluster‐containing DHAD from the cyanobacterium *Synechocystis sp*. PCC 6803 (*Sn*DHAD).^[26]^ As purified, its catalytic rate for the reaction with DHIV is *k*_cat_ ∼0.47 s^−1^.^[26]^ Metal analysis indicated that the Fe content per protein monomer is only 0.62 atoms. The enzyme was thus anaerobically incubated with sodium sulfide and ammonium ferrous sulfate, which led to an increase in the Fe content to 2.29 atoms per monomer, suggesting the presence of an active site with a fully loaded [2Fe−2S] cluster. However, despite this increase in Fe loading the activity of *Sn*DHAD dropped to 30 % of the value recorded for enzyme before its incubation.^[26]^

We suspected that the low activity of *Sa*DHAD and *Cj*DHAD may be due to the absence of functional Fe−S clusters after purification. We therefore determined their iron content using inductively‐coupled plasma optical emission spectrometry (ICP‐OES). *Sa*DHAD and *Cj*DHAD contain sub‐stoichiometric amounts of iron, 0.30 and 0.22 atoms per protein monomer, respectively (Table 1). This suggests that functional Fe−S clusters are indeed absent in *Sa*DHAD and *Cj*DHAD when initially purified, in correlation with the minimal activity of these enzymes. However, there is a large difference between the enzymes in this study and *Sn*DHAD (see preceding paragraph). While the iron content of these three enzymes as purified is comparable, their catalytic proficiency differs by several orders of magnitude. This observation indicates that other factors, for instance the nature of the Fe−S clusters (i.e. [2Fe−2S] vs. [4Fe−4S]) and their oxidation state(s), may contribute to both the activity and stability of these enzymes.

**Table 1:** Fe content and catalytic rates (s^−1^) of *Sa*DHAD and *Cj*DHAD as initially purified and after activation under aerobic or anaerobic conditions. Catalytic rates were determined with 100 nM *Sa*DHAD and *Cj*DHAD using 5 mM DHIV at 37 °C.

Enzyme	Activation method	Fe atoms per subunit	Rate [*k*_cat_ s^−1^]
*Sa*DHAD	None	0.30±0.02	7.6×10^−4^
	Aerobic	4.66±0.57	1.8±0.1
	Anaerobic	5.45±0.31	1.0±0.0
*Cj*DHAD	None	0.22±0.07	3.4×10^−4^
	Aerobic	4.71±0.01	3.8±0.6
	Anaerobic	5.94±0.19	6.7±0.4

### Enzyme activation

To activate *Sa*DHAD and *Cj*DHAD, we modified a protocol previously developed for the activation of *Ss*DHAD^[30]^ and incubated both enzymes for one hour (aerobically or anaerobically) with the reducing agents, sodium dithionite and 2‐ME, in the presence of ammonium ferrous sulfate in 50 mM HEPES, pH 8, at 37 °C. The optimal concentrations for the reducing agents and Fe^2+^ were established by comparing the effect of three different mixtures on the catalytic activities of *Sa*DHAD and *Cj*DHAD (Figure S5). The mixture with 50 mM sodium dithionite, 200 mM 2‐mercaptoethanol and 10 mM (NH_4_)_2_Fe(SO_4_)_2_ resulted in the highest catalytic activity and was thus used for all subsequent activations of the enzymes. Both the aerobic and anaerobic activation procedures led to a significant increase in both the iron content and catalytic activity (Figure 4 and Table 1). These results suggest that *Sa*DHAD and *Cj*DHAD are fully activated with reconstituted Fe−S clusters, likely of the [4Fe−4S] form (discussed below).

### Stability of activated *Sa*DHAD and *Cj*DHAD under aerobic conditions

**Figure 5:**
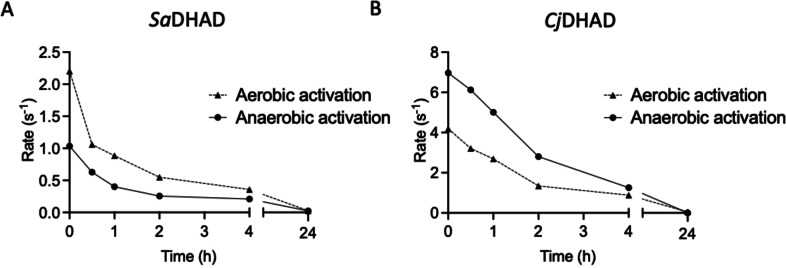
Stability of *Sa*DHAD (A) and *Cj*DHAD (B) after activation in an aerobic environment at 25 °C. Enzyme and substrate (DHIV) concentrations were 100 nM and 5 mM, respectively.

In an aerobic environment at room temperature, activated *Sa*DHAD and *Cj*DHAD rapidly lost activity (Figure 5). Irrespective of how the enzymes were activated (aerobic or anaerobic), their activities dropped by at least 30 % after one hour with only residual activity left after 24 h. The Fe content of each enzyme sample was re‐measured at the end of the 24‐hour incubation period. For the anaerobically activated enzymes, the metal content decreased modestly (*Sa*DHAD: from 5.45±0.31 to 5.03±0.06 atoms per monomer; *Cj*DHAD: from 5.94±0.19 to 4.33±0.12 atoms per monomer), however it decreased more significantly for the aerobically activated enzymes (*Sa*DHAD: from 4.66±0.57 to 1.14±0.02 atoms per monomer; *Cj*DHAD: from 4.71±0.01 to 1.95±0.06 atoms per monomer). It is thus likely that anaerobic and aerobic activations lead to Fe−S clusters with different oxidation states, with the more oxidized form (i.e. after aerobic activation) being more labile. However, it is interesting to point out that the oxidation states of these clusters do not appear to correlate to the activity of the two DHADs; the more oxygen‐exposed cluster (aerobically activated) is more active in *Sa*DHAD, whereas the less oxygen‐exposed cluster (anaerobically activated) is more active in *Cj*DHAD (Figure 5). The enhanced lability of the Fe−S clusters when activated aerobically, but the lack of a correlation between the mode of activation (aerobic vs. anaerobic) and activity in the two enzymes suggest that the oxidation state of the clusters affects their stability significantly but is of lesser relevance to their catalytic efficiency. This interpretation is also consistent with the rapid inactivation that was also observed for anaerobically purified *Ec*DHAD (∼50 % loss of activity after 30 minutes at 25 °C in an aerobic environment), an enzyme with a confirmed oxygen‐sensitive [4Fe−4S] cluster.[^11^, ^40^]

### Characterisation of the catalytic properties of activated *Sa*DHAD and *Cj*DHAD

Both the aerobic and anaerobic activation procedures led to large increases in activity and Fe content for both enzymes (Figure 4 and Table 1). For *Sa*DHAD the corresponding values are a ∼15 and ∼18‐fold increase in iron loading, and ∼2,400‐ and ∼1,300‐fold enhancement of the rate. For *Cj*DHAD, the aerobic and anaerobic treatments led to a ∼21 and ∼27‐fold increase in the iron loading, respectively, and a concomitant ∼11,100‐ and ∼19,700‐fold enhancement of the catalytic rate. Thus, while there is a correlation between the iron loading and the activity, the two enzymes respond again differently to aerobic and anaerobic treatments (compare to previous paragraph); while the anaerobic incubation leads to higher loading in both enzymes, only in *Cj*DHAD does this also lead to an increased rate (nearly two‐fold). In *Sa*DHAD, the rate after anaerobic treatment reaches only ∼60 % of that after aerobic treatment despite a ∼20 % increase in iron loading. Since both enzymes were treated identically, the observed difference after anaerobic incubation supports the hypothesis that these enzymes may indeed prefer different oxidation state(s) for optimal activity.

In order to assess if the activation of the two enzymes affects their interaction with the substrate (DHIV), reaction rates were measured at various concentrations of substrate and analysed using Michaelis‐Menten kinetics (Figure 4; data were only measured for the most active forms of the enzymes, i.e. the aerobically activated *Sa*DHAD and the anaerobically activated *Cj*DHAD). For both the enzymes as purified and activated, saturation‐type behaviour for DHIV is observed. While the activation procedure greatly impacts on the k_cat_ values, its effect on the K_m_ values is minimal. For *Sa*DHAD the K_m_ value slightly increases from 0.93 mM to 1.1 mM upon activation whereas for *Cj*DHAD virtually no difference is observed. Thus, substrate binding in both DHADs is largely unaffected by the activation procedure, indicating that minimal structural changes occurred during this process. Furthermore, the catalytic parameters of activated *Sa*DHAD and *Cj*DHAD are in good agreement with those of other known DHADs (Table 2).

**Table 2:** Comparison of the kinetic parameters of several DHADs for their reaction with DHIV.

Organism (DHAD)	K_m_ [mM]	k_cat_ [s^−1^]	k_cat_/K_M_ [M^−1^s^−1^]	Nature of Fe−S cluster
*S. aureus* (*Sa*DHAD)	1.1	2.1	1909	[4Fe−4S]
*C. jejuni* (*Cj*DHAD)	0.82	6.5	7927	[4Fe−4S]
*E. coli* (*Ec*DHAD)^[11]^	1.5	70	46,667	[4Fe−4S]
M. tuberculosis (*Mt*DHAD)^[28]^	2.0	1.87	935	[2Fe−2S]
*A. thaliana* (*At*DHAD)^[29]^	5.7	1.2	210	[2Fe−2S]
*S. oleracea* (*So*DHAD)^[10]^	1.5	25	16,667	[2Fe−2S]
*Synechocytis* (*Sn*DHAD)^[26]^	0.14	0.47	3,357	[2Fe−2S]
*S. solfataricus* (*Ss*DHAD)^[30]^	2.1	0.32	152	[2Fe−2S]

### pH profile and thermostability

**Figure 6:**
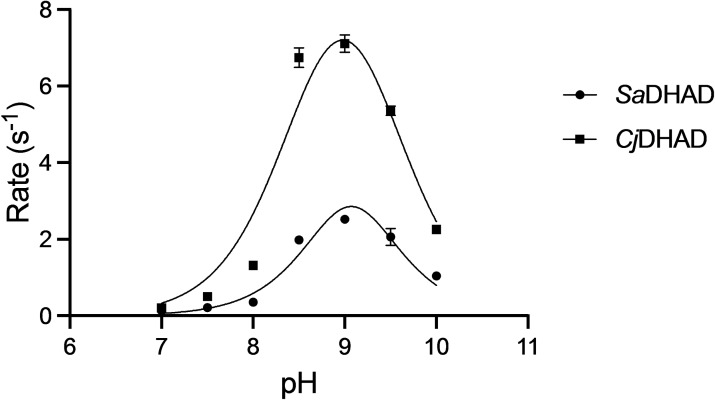
The catalytic activities of SaDHAD (circles) and CjDHAD (squares) were determined at various pH values (at 37 °C) using 50 mM HEPES (pH 7–7.5), Tris‐HCl (pH 8–9) and Glycine buffers (pH 9.5–10). Enzyme and substrate concentrations were 100 nM SaDHAD, 50 nM CjDHAD and 5 mM DHIV. The data were fit to an equation derived for a diprotic system.

Catalytic assays with the substrate DHIV were performed at different pH values ranging from 7 to 10 using three different buffers with activated *Sa*DHAD and *Cj*DHAD to determine the effect of pH on their activities (Figure 6). Both enzymes perform optimally at pH 9, slightly higher than the optimal pH of ∼8 that has been observed in previously characterised DHADs and DHTs from organisms such as *S. solfataricus, M. tuberculosis, Methanococcus* spp. and *R. leguminosarum*.[^28^, ^41^, ^42^, ^43^] The pH profiles of the two enzymes were fit to an equation derived for a diprotic system,[^44^, ^45^, ^46^] which provides estimates for the two relevant protonation equilibria (p*K*_a1_ and p*K*_a2_) that affect the catalytic rates of *Sa*DHAD and *Cj*DHAD. For both enzymes these p*K*_a_ values are very similar (*Sa*DHAD: 8.75 and 9.40; *Cj*DHAD: 8.40 and 9.55). The assignment of these p*K*_a_ values to respective residues in the active site is inherently difficult, and in the absence of structural and mutagenesis data it remains speculative. Nonetheless, the sharp increase in the activity as the pH is increased from 7.0 to 9.0 (corresponding to p*K*_a1_) is consistent with the deprotonation of Ser489, the residue proposed to initiate catalysis by abstracting a proton from the C2 position of the substate (Figures 2 and 3). The sharp decline of the activity between pH 9.0 and 10.0 (corresponding to p*K*_a2_) may be due to the reduced ligand exchange rate of the hydroxide bound to the Fe−S cluster after the cleavage of the C3−OH (Figure 2). The p*K*_a_ value of a water molecule bound to Fe^2+^ is consistent with this assignment but it needs to be pointed out that no concrete evidence about the oxidation state(s) of the Fe−S clusters in *Sa*DHAD and *Cj*DHAD is currently available (see also below). However, despite the uncertainties in the assignment of these p*K*_a_ values the experimental data support the recently proposed model for the reaction mechanism employed by DHADs (Figure 2).

**Figure 7:**
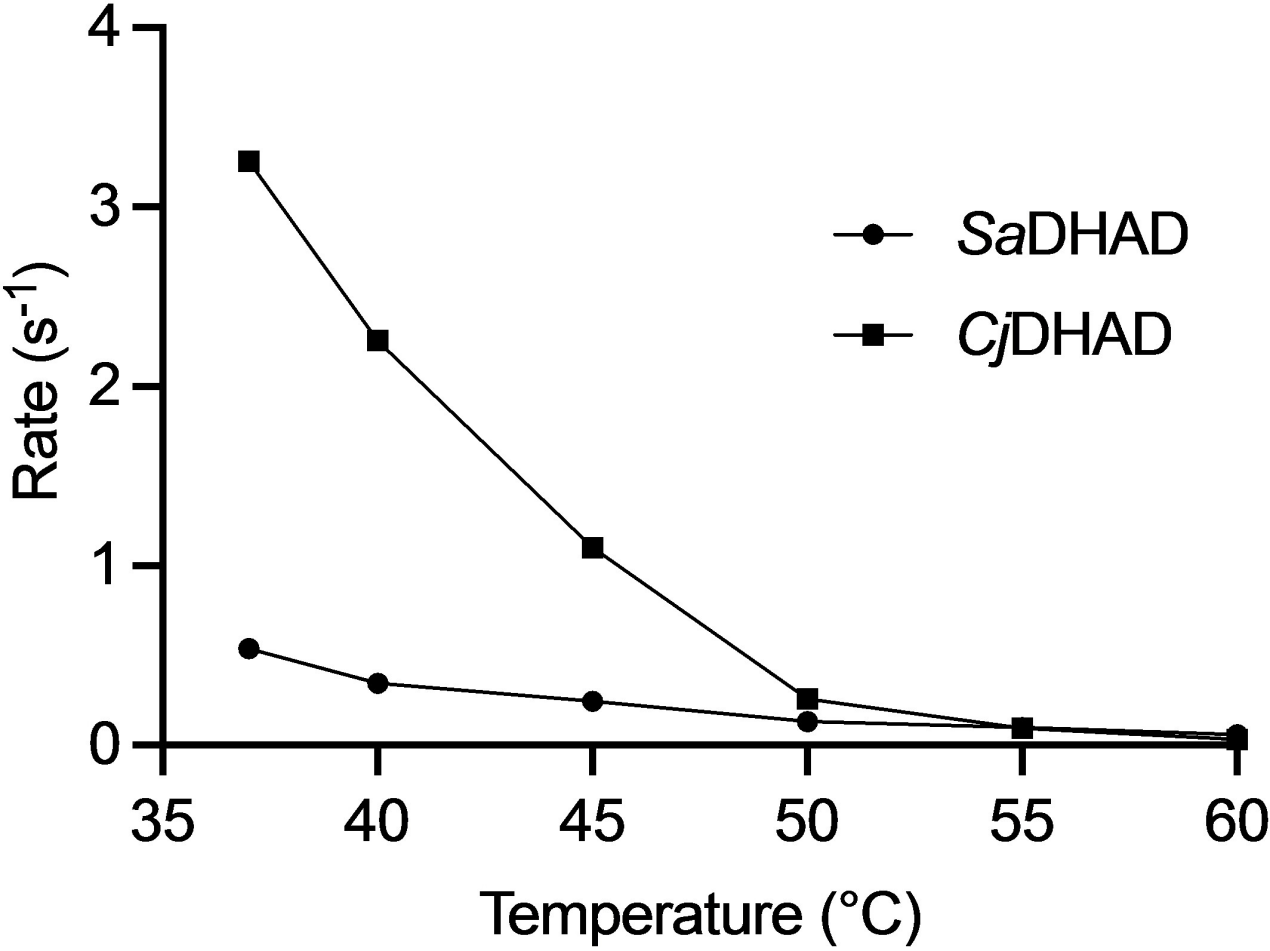
Dependence of the catalytic activity of activated SaDHAD (circles) and CjDHAD (squares) on temperature. Enzyme samples were pre‐incubated for 10 min at specific temperatures before the reactions were initiated by addition of the substrate. Enzyme and substrate concentrations were 100 nM SaDHAD, 50 nM CjDHAD and 5 mM DHIV in 50 mM Tris‐HCl, pH 8.5.

The thermostability of *Sa*DHAD and *Cj*DHAD was also assessed with kinetic measurements after 15‐minute incubation at temperatures ranging from 37 °C to 60 °C (Figure 7). After incubation, the enzymes were assayed at 37 °C, the standard temperature used to assay DHAD activity. Activities sharply decrease for both enzymes, with virtually no activity measurable for samples incubated at 60 °C. The ∼30 % drop in activity between the narrow temperature interval from 37 °C to 40 °C may indicate that the inactivation of *Cj*DHAD and *Sa*DHAD is likely to be due to the instability of the Fe−S cluster, not that of the protein itself, as also suggested for other DHADs in a previous study.^[12]^

### Structure of the Fe−S cluster in *Sa*DHAD and *Cj*DHAD

**Figure 8:**
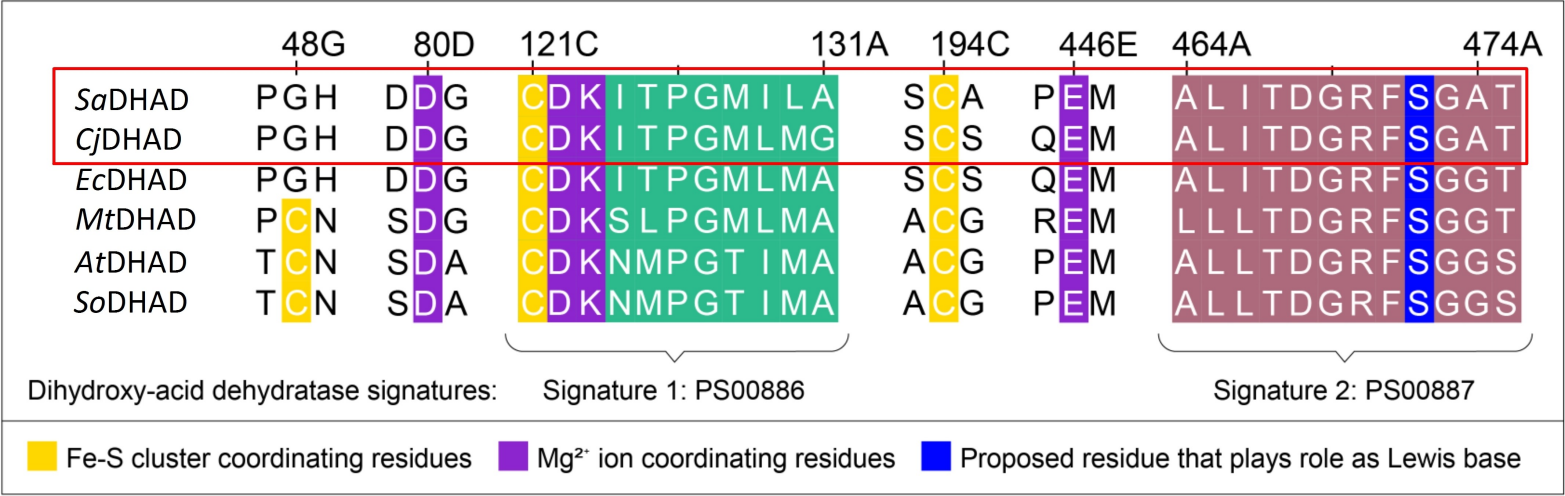
Multiple sequence alignment of DHADs from *S. aureus* (*Sa*DHAD), *C. jejuni* (*Cj*DHAD)*, E. coli* (*Ec*DHAD)*, M. tuberculosis* (*Mt*DHAD)*, A. thaliana* (*At*DHAD) and *S. oleracea* (*So*DHAD). The two signature motifs (PS00886 and PS00887 as indicated by PROSITE^[47]^) are highlighted, as are several residues known to play an important role in the function of DHADs.

The combined data presented above suggest that both *Sa*DHAD and *Cj*DHAD contain oxygen‐sensitive [4Fe−4S] clusters in their active sites. Unfortunately, diverse attempts to crystallise these enzymes (as purified and after activation) failed, possibly a consequence of the instability of their Fe−S clusters (and the associated inhomogeneity of the protein samples). We thus performed a detailed sequence comparison between *Sa*DHAD and *Cj*DHAD and DHADs with well‐established Fe−S clusters, i.e. *Ec*DHAD with a [4Fe−4S] cluster and *Mt*DHAD, *At*DHAD and *So*DHAD that have [2Fe−2S] clusters in their active sites (Figure 8). Two signature motifs from the IlvD/EDD family are present in all DHADs. The first is located at the N‐terminus and consists of 11 amino acids from sequence alignment position 121 to 131. Cysteine residue 121 is strictly conserved among all DHADs and provides a ligand for one of the irons in the Fe−S cluster (Figure 3; Cys121 in the alignment corresponds to Cys139 in the sequence of *At*DHAD). This motif also contains two of the invariant ligands for the catalytically essential Mg^2+^ ion, i.e. Asp122 and the carboxylated Lys123 (Asp140 and Lys141 in *At*DHAD; Figure 3).

The second signature motif in DHADs is located at the C‐terminus and spans alignment residue positions 464 to 475 (Figure 8). Contained within this motif is an invariant serine residue at position 472 (Ser489 in *At*DHAD; Figure 3). This residue has been proposed to act as the Lewis base during catalysis (Figure 2).[^10^, ^25^, ^48^] Other essential residues important for the function of DHADs are distributed across the protein sequence, including two additional cysteine ligands for the iron ions in the Fe−S clusters (Cys48 and Cys194, i.e. Cys66 and Cys211 in *At*DHAD) and two ligands for the Mg^2+^ ion (Asp80 and Glu446, i.e. Asp98 and Glu463 in *At*DHAD). However, it should be noted that the cysteine residue in alignment position 48 (Figure 8) is only conserved in DHADs with known [2Fe−2S] clusters[^23^, ^24^, ^25^, ^28^, ^29^] while in DHADs with a [4Fe−4S] cluster, notably *Ec*DHAD,^[11]^ a glycine residue resides in the corresponding location. Both *Sa*DHAD and *Cj*DHAD also have a glycine residue in this position (Figure 8) thus adding further evidence to the presence of a [4Fe−4S] cluster in these bacterial enzymes.

**Figure 9:**
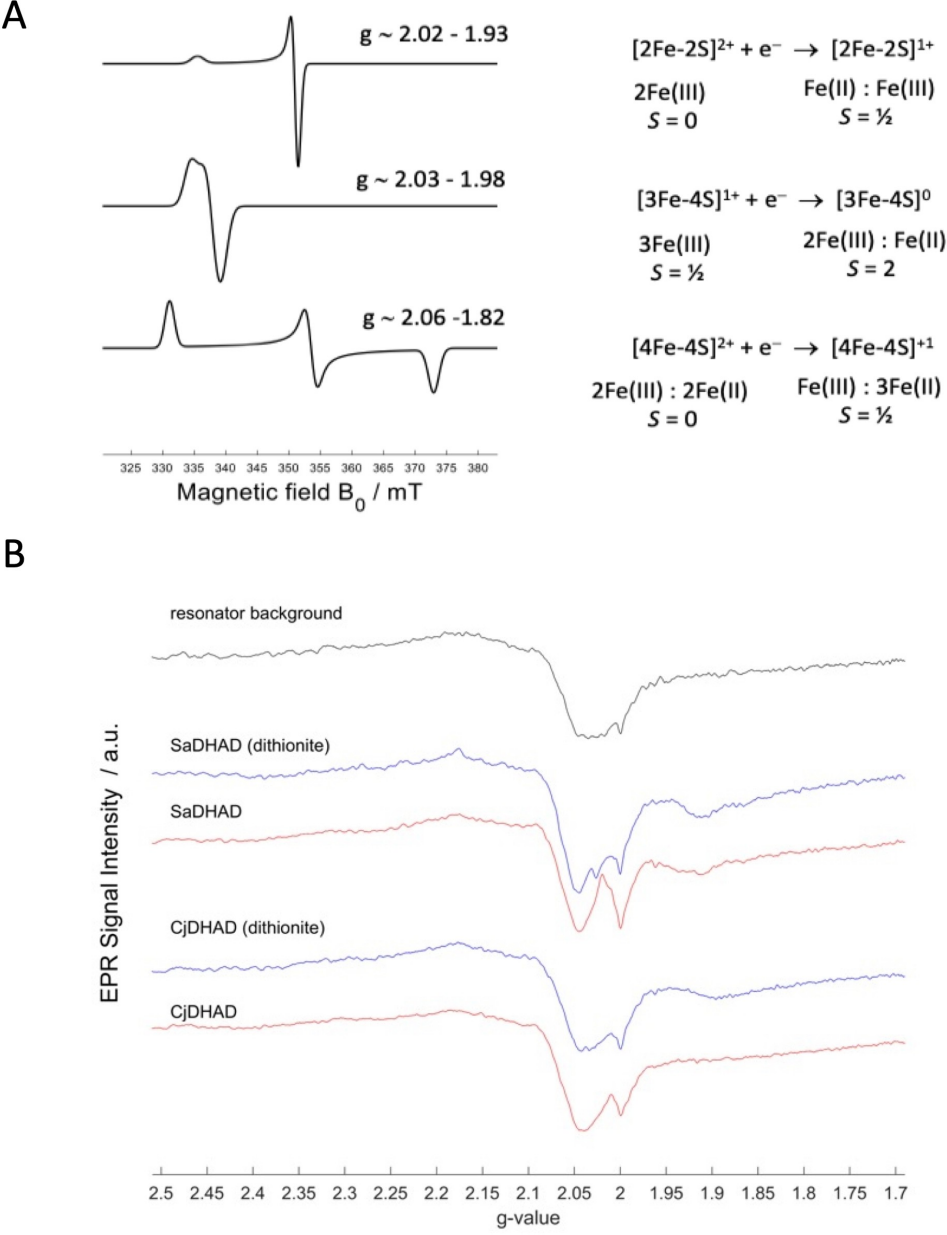
(A) Simulated X‐band CW EPR spectra of different Fe−S clusters.[^54^, ^55^] (B) X‐band CW EPR spectra at 10 K of anaerobically activated *Sa*DHAD and *Cj*DHAD with and without sodium dithionite.

Since it has not yet been possible to visualise the three‐dimensional structures of a [4Fe−4S] cluster in a DHAD, we employed electron paramagnetic resonance (EPR) and spectro‐electrochemical measurements to gain further insight into the identity of the Fe−S clusters in *Sa*DHAD and *Cj*DHAD. Analysis of EPR spectra from Fe−S clusters frequently allow for distinction between two‐iron, three‐iron and four‐ion clusters. Examples are illustrated in Figure 9A. Although the majority of Fe−S cluster containing enzymes play roles in electron transfer reactions, DHADs and DHTs are not the only examples of such enzymes that perform non‐redox‐type reactions. Aconitase, for instance, catalyses the stereo‐specific isomerisation of citrate to isocitrate in the Krebs cycle.^[49]^ It can accommodate a [4Fe−4S] cluster, similar to *Ec*DHAD and possibly *Sa*DHAD and *Cj*DHAD, but these clusters have proven to be mostly EPR silent; either they are in a diamagnetic spin state or a paramagnetic spin state with resonances beyond the field and frequency range of routine EPR spectrometers.[^50^, ^51^, ^52^] Indeed, aerobically and anaerobically activated *Cj*DHAD is EPR silent (at 10 K, 9.38 GHz over the field range 0–1T), irrespective of the absence or presence of a reducing agent (Figure 9B). CW EPR spectra collected for *Sa*DHAD under identical conditions contain only extremely weak signals consistent with the presence of a mixture of paramagnetic species at low concentration, probably indicating stages of cluster degradation (compare Figures 9A and 9B). The presence of a reducing agent produces no significant change to the EPR spectra. It thus appears that at least in *Sa*DHAD, the Fe−S cluster quite rapidly disintegrates, consistent with the relatively rapid loss of catalytic activity (Figure 5).

Similar to the EPR spectra, the redox potentials of Fe−S clusters can vary significantly depending on the type of cluster that is present in the active site of an enzyme. For clusters of the [2Fe−2S] type potentials may vary between 0 and −0.4 V, while for [3Fe−4S] and [4Fe−4S] clusters potentials in the +0.2 V to −0.4 V and −0.2 V to −0.7 V range have been reported.^[53]^ In order to assess the potential of Fe−S cluster of *Cj*DHAD, spectroelectrochemical measurements (300 nm to 1000 nm) were performed with an anaerobically activated sample (Figure S6). Minimal changes in the UV‐vis spectra were observed at potentials as low as −0.75 V vs. Ag/AgCl (i.e. −0.55 V vs. NHE). Overall, the spectral changes observed by sweeping from high to low potentials are insignificant. The peak at 410 nm, characteristic of an oxidised [4Fe−4S]^2+^ cluster, persists throughout the experiments. During the course of the experiment, some loss of the Fe−S cluster via irreversible degradation was observed. Therefore, *Cj*DHAD and *Sa*DHAD, like aconitase, are not required to be redox active for catalysis. Indeed, reduction of the Fe−S cluster can lead to irreversible degradation of the active site.

### Enzyme inhibition

**Figure 10:**
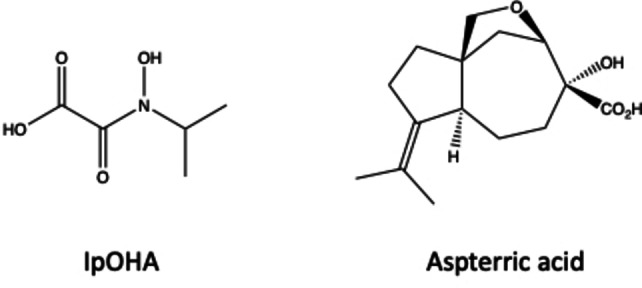
Chemical structures of the known KARI inhibitor, IpOHA, and DHAD inhibitor, aspterric acid.

**Figure 11:**
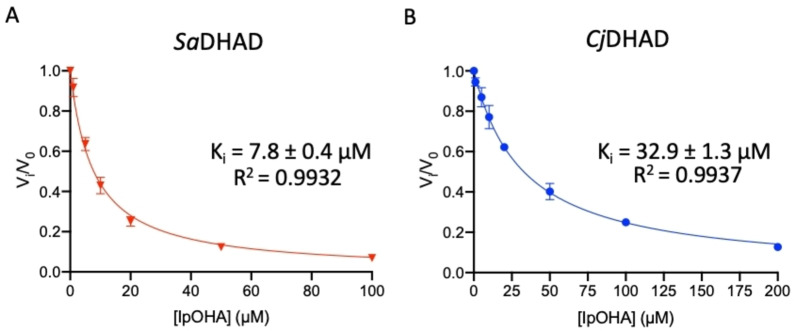
Inhibition of SaDHAD and CjDHAD by IpOHA (after 30‐min pre‐incubation).

DHAD, similar to other enzymes of the BCAA pathway (i.e., AHAS and KARI), is a suitable target for herbicides and antimicrobial agents.[^56^, ^57^, ^58^] N‐isopropyloxalyl hydroxamate (IpOHA; Figure 10), for instance, is a potent time‐dependent inhibitor of KARI, the enzyme preceding DHAD in the BCAA pathway (Figure 1), with *K*_i_ values in the nM range.[^6^, ^8^, ^21^, ^59^, ^60^] For both *Sa*DHAD and *Cj*DHAD, IpOHA also acts as a slow‐binding inhibitor. In an assay containing 5 mM DHIV and 100 μM IpOHA, the inhibition increased from 2.1 % to 91.9 % upon pre‐incubation for *Sa*DHAD. The corresponding values for *Cj*DHAD are 4.8 % and 75.8 %. Using pre‐incubated samples, the *K*_i_ values of *Sa*DHAD and *Cj*DHAD for IpOHA were determined to 7.8 μM and 32.9 μM, respectively (Figure 11). Thus, IpOHA is a good inhibitor for DHADs albeit not as effective as for KARI (with *K*_i_ values ranging in the nM range[^6^, ^21^, ^59^, ^60^]). This is not surprising as IpOHA has been described as a transition state analog of KARI that resembles the substrate‐bound rather than the product‐bound state.^[21]^

**Figure 12:**
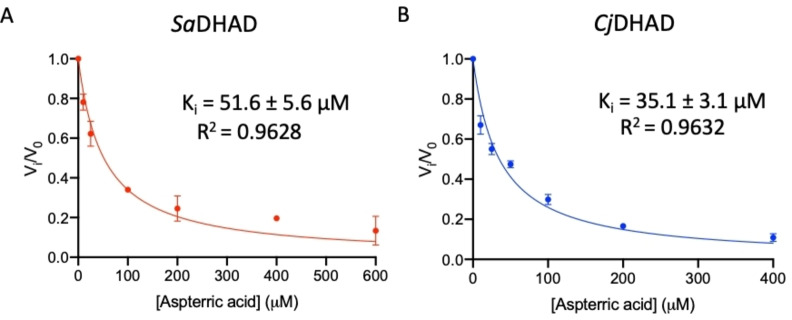
Inhibition of SaDHAD and CjDHAD by aspterric acid.

Inhibition studies with aspterric acid (Figure 10), a known natural inhibitor of DHAD,[^28^, ^29^] determined *K*_i_ values of 51.6 μM and 35.1 μM for *Sa*DHAD and *Cj*DHAD, respectively (Figure 12). Aspterric acid has been tested against DHADs from plant and bacteria in recent studies[^26^, ^28^, ^29^] and was shown to be most potent for cyanobacterial DHAD (*Sn*DHAD; *K*_i_=9 nM).^[26]^ It was also shown to significantly inhibit both *At*DHAD (*K*_i_=0.3 μM) and *Mt*DHAD (*K*_i_=10.1 μM).[^28^, ^29^] It is, however, currently unknown why the inhibitory potency of aspterric acid varies by three orders of magnitude between different DHADs. The nature of the Fe−S cluster alone may not account for this variation since *Sn*DHAD, *At*DHAD and *Mt*DHAD contain [2Fe−2S] clusters, and *Sa*DHAD and *Cj*DHAD contain [4Fe−4S] clusters (although the enzymes with the less complex [2Fe−2S] clusters are generally stronger affected, possibly because their active sites are either more open or more flexible). In summary, both IpOHA and aspterric acid are suitable lead compounds for the development of novel anti‐microbial drugs that target bacterial DHADs. IpOHA derivatives that may bypass the time‐dependence of their interactions with KARI are currently being designed and may also become relevant as DHAD inhibitors.[^18^, ^20^, ^22^]

## Conclusions

DHAD, along with other enzymes from the BCAA pathway (Figure 1), is an attractive target for novel anti‐microbial drug discovery. DHAD is still a relatively poorly characterised enzyme, and thus the characterisation of *SaDHAD* and *Cj*DHAD provides significant functional and catalytic insight into these DHADs. A multiple sequence alignment of *Sa*DHAD and *Cj*DHAD with previously characterised DHADs suggests that they are likely to contain a [4Fe−4S] cluster like the DHAD from *E. coli* (*Ec*DHAD). The Fe−S cluster‐coordinating residues Cys‐121 and Cys‐194 and the Mg^2+^ ion coordinating residues Asp‐80, Asp‐122, Lys‐123 and Glu‐446 are fully conserved in all DHADs, but the DHADs with a [4Fe−4S] cluster lack a cysteine in alignment position 48 (Figure 8). Recombinant *Sa*DHAD and *Cj*DHAD have been successfully expressed and purified from *E. coli* BL21 cells. Initial kinetic studies showed both enzymes to be virtually inactive, which was partially due to the lack of Fe−S cluster loading as indicated by ICP‐OES analysis (Table 1). Reconstitution of the cluster by incubation with both Fe^2+^ and reducing agents did not only increase the Fe−S loading of these enzymes, but also led to a dramatic increase in their catalytic rates (Figure 4). Stability experiments showed, however, that the activated enzymes lost their activity after 24 h under aerobic conditions at room temperature (Figure 5) but that activity can be preserved long term by storing the enzymes at −80 °C. The pH profiles for *Sa*DHAD and *Cj*DHAD indicate that optimal activity is reached at a pH of ∼9 (Figure 6), slightly higher than the optima reported for other DHADs, but the catalytic mechanism is likely to be conserved. Furthermore, the thermostability of both *Sa*DHAD and *Cj*DHAD sharply decline at temperatures above 37 °C; at 60 °C the enzymes are essentially inactive (Figure 7). Thus, the sensitivity to inactivation by oxygen and elevated temperatures render both *Sa*DHAD and *Cj*DHAD as unsuitable for biotechnological processes that aim to exploit the catalytic potential of enzymes of the BCAA pathway to manufacture diverse platform chemicals (e.g. isobutanol[^12^, ^13^, ^31^, ^32^]). However, both *Sa*DHAD and *Cj*DHAD (and other bacterial DHADs) are suitable targets for the development of much needed new chemotherapeutics to treat infectious diseases. Catalytic measurements (Figures 11 and 12) have shown that IpOHA, a KARI inhibitor, and aspterric acid, a recently described DHAD inhibitor (Figure 10), inhibit both *Sa*DHAD and *Cj*DHADs potently with *K*_i_ values in the low micromolar range. These compounds thus provide excellent starting points to further develop leads for novel treatments for infectious diseases. For instance, variants of IpOHA have recently been reported to be significantly more potent towards KARI from *M. tuberculosis* and similar modifications may thus also enhance the inhibitory potency of this compound for DHADs.^[22]^

## Materials and Methods

### Transformation and expression trial

The DHAD genes (ilvD) of *Sa* and *Cj* with hexa‐histidine tag at the N‐terminal of the polypeptide were cloned into pET‐28a vector using *NdeI* and *XhoI* restriction sites and ordered from Gene Universal. The construct was transformed into *E. coli* BL21 cells using 100 μg/mL kanamycin plates. Single colonies were taken and inoculated in media for starter cultures and glycerol stock. For the expression trials, the protein was expressed in 1 mL autoinduction media. The cells were lysed and centrifuged at 14,500 rpm. The supernatant and the pellet samples were run on an SDS‐PAGE gel to observe the presence of the proteins of interest.

### Protein expression and purification

The inoculated cell culture was grown at 37 °C in autoinduction media (ZYP‐5052) containing 100 μg/mL kanamycin for three hours after which the temperature was decreased to 18 °C and the protein was expressed overnight. The cells were harvested by centrifugation at 4000 rpm at 4 °C for 30 minutes and was stored at −80 °C until purification. The cell paste was resuspended in binding buffer with protease inhibitor cocktail, DNAse and lysozyme and was lysed by sonication. The binding buffer contained 300 mM NaCl, 20 mM imidazole, and 50 mM HEPES pH 8. The cell lysate was centrifuged at 4 °C for one hour at 18000 RPM and the supernatant was loaded onto ÄKTA FPLC system. The protein was purified through immobilized metal affinity chromatography (IMAC) using 20 mL HisPrep Fast Flow 16/10 Ni‐NTA‐agarose resin column. The elution buffer used to elute the protein was same as the binding buffer except for the concentration of imidazole, which was increased to 300 mM. Next, the protein was further purified by size exclusion chromatography using a HiPrep 26/60 Sephacryl S‐300 HR column with a buffer containing 10 % glycerol and 50 mM HEPES pH 8. The protein concentration was quantified from its absorbance at 280 nm using its theoretical extinction coefficient calculated with Prot‐param. TGX FastCast Acrylamide Kit from Bio‐Rad was used to prepare all polyacrylamide gels. Precision Plus Protein Unstained Ladder was used to establish the standards. The gels were run with 1x running buffer at 110 V for 75 minutes. The gels were stained using Coomassie Instant Blue and destained with distilled water.

### Synthesis of DHIV

**Scheme 1:**
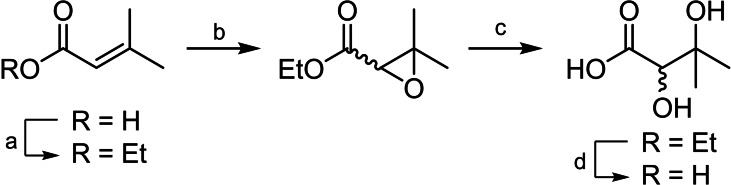
(a) Ethanol, H_2_SO_4_, reflux, 36 h, 98 %. (b) mCPBA, CHCl_3_, reflux, 17 h, 45 %. (c) HCl, H_2_O/THF, rt, 17 h, 56 %. (d) NaOH, THF/H_2_O, rt, 17 h, quant.

Various syntheses have been reported for the formation of the DHAD substrates 2,3‐dihydroxy‐3‐methylpentanoic acid (DHIV) or its derivative 2,3‐dihydroxy‐3‐methylpentanoic acid (DHMV).[^48^, ^61^, ^62^, ^63^, ^64^, ^65^, ^66^, ^67^] Here, DHIV was synthesised in 25 % yield across four steps from 3‐methylbut‐2‐enoic acid (Scheme 1), similarly to the method reported by Chunduru et al.^[62]^ Details for the full synthesis are described in the Supporting Information.

### Enzyme assay and characterisation

For the enzyme assay, a modified version of the dinitrophenylhydrazine (DNPH) colorimetric endpoint assay described by Kiritani and Wagner^[68]^ and Katsuki et al.^[69]^ was used. DNPH was purchased from Sigma and 1.6 mg/mL was prepared in 2 N HCl. All assays were done at 37 °C unless stated otherwise. A typical master reaction had a total volume of 2 mL and the reaction was started by adding dihydroxy‐isovalerate (DHIV). Aliquots (380 μL) were taken from master reaction at each time point. The aliquot reaction was stopped by adding 0.1 volume (38 μL) of trichloroacetic acid (TCA) and was centrifuged at 14,500 rpm for 5 minutes to remove precipitation. Aliquot (360 μL) from the supernatant was taken and incubated with 120 μL of 1.6 mg/mL DNPH for 15 minutes at room temperature until 540 μL of 2 M NaOH was added. After 5 minutes, the formation of the hydrazone complex formed by the reaction of keto‐acid with the hydrazine group of DNPH was measured spectrophotometrically at 430 nm. The amount of keto product was calculated from a standard curve that was plotted using KIV, which was purchased from Sigma. Michaelis‐Menten plots were fitted using GraphPad Prism.

### Activation and stability of DHAD

In order to activate DHAD, a modified protocol from Carsten et al.^[30]^ was used. 50 mM sodium dithionite, 200 mM 2‐mercaptoethanol (2‐ME) and 10 mM Fe^2+^ from ammonium ferrous sulfate were added to 500 μL of enzyme solution to make up a total volume of 3 mL. The solution was incubated at 37 °C for 1 h before being buffer exchanged into 10 % glycerol, 50 mM HEPES pH 8 solution using a 10DG Econo desalting columns from Bio‐Rad and concentrated for further experiments. The activated enzymes stored at −80 °C retained stable activity for at least seven days (Figure S7). For the stability of activated *Cj* and *Sa*DHADs, the activity was measured immediately after activation (t=0), then after 30 minutes, one, two, four and 24 h. The activity was measured using the DNPH as described previously with 200 nM enzyme, 5 mM substrate, 5 mM MgCl_2_ and 50 mM Tris‐HCl, pH 8.5.

### pH profile and thermostability

*Sa*DHAD and *Cj*DHAD were assayed with pH ranging from 7.0 to 10.0 to determine its pH profile. For pH 7.0 to 8.0 HEPES, for 8.0 to 9.0 Tris‐HCl and for 9.5 to 10 glycine buffers were used. For the thermostability experiment, the enzyme was preincubated in the assay solution at said temperature for 15 minutes. Then the substrate was added and the reaction was monitored at 37 °C with timepoints taken every five minutes. The rate was determined using the DNPH assay with 100 nM *Sa*DHAD (50 nM *Cj*DHAD), 5 mM substrate, 5 mM MgCl_2_ and 50 mM of Tris‐HCl pH 8.5.

### EPR and spectroelectrochemistry of *Sa*DHAD and *Cj*DHAD

X‐band CW EPR measurements were carried out at 10 K (Bruker E500 spectrometer equipped with a He closed cycle cryostat) on ∼50 μM/100 μL of activated *Sa* and *Cj*DHADs. Sodium dithionite was added to the reduced samples to a concentration of 200 μM. Spectroelectrochemistry was carried out (under nitrogen) with ∼50 μM of anaerobically activated *Cj*DHAD at 10 °C. Spectra were measured down to −0.75 V vs. Ag/AgCl (−0.55 V vs. NHE) with a Pine Instruments honeycomb Au spectroelectrochemical electrode and thin layer cell. Mediators for electron transfer were the complexes [Co(AMMEN_5_Ssar)]^3+^, [Co(sep)]^3+^, [Co(AMMEsar)]^3+^, [Co(cis‐diammac)]^3+^ and [Co(trans‐diammac)]^3+^, all at a concentration of 20 μM.^[70]^ The experiments were carried out under a constant blanket of nitrogen over the top of the cell.

### Inhibition of DHAD

The inhibition of *Sa*DHAD and *Cj*DHAD by IpOHA and aspterric acid was evaluated using the DNPH assay. The reaction mixture contained 600 nM *Sa*DHAD (100 nM *Cj*DHAD), 5 mM DHIV, 5 mM MgCl_2_ in 50 mM Tris‐HCl pH 8.5. Inhibition of IpOHA was determined with 30‐minute preincubation before starting the reaction with the addition of the substrate. Concentrations between 0 to 200 μM of IpOHA were used to determine the *K_i_* value. For assays with aspterric acid, no pre‐incubation was applied and the inhibitor concentration ranged from 0 to 600 μM. The inhibition constants for IpOHA and aspterric were calculated using the simple inhibition equation (Equation [1]).

(1) $$V_{i}=\frac{V_{u}}{\left(1+\;\frac{\left[I\right]}{K_{i}}\right)}$$

where V_i_ is the rate at the inhibitor concentration [I], V_u_ is the uninhibited rate and *K_i_* is the inhibition constant.

## Conflict of interest

The authors declare no conflict of interest.

1

## Supporting information

As a service to our authors and readers, this journal provides supporting information supplied by the authors. Such materials are peer reviewed and may be re‐organized for online delivery, but are not copy‐edited or typeset. Technical support issues arising from supporting information (other than missing files) should be addressed to the authors.

Supporting Information

## Data Availability

The data that support the findings of this study are available from the corresponding author upon reasonable request.
