# Supplementary material for: Dihydroxy‐Acid Dehydratases From Pathogenic Bacteria: Emerging Drug Targets to Combat Antibiotic Resistance
Source: Chemistry. 2022 Jun 16;28(44):e202200927. doi: 10.1002/chem.202200927 (PMC9543379; doi:10.1002/chem.202200927)
Supplement: Supplementary file 1 — Supporting Information [file CHEM-28-0-s001.pdf]

Chemistry–A European Journal

Supporting Information

Dihydroxy-Acid Dehydratases From Pathogenic Bacteria: Emerging Drug Targets to Combat Antibiotic Resistance

Tenuun Bayaraa, Jose Gaete, Samuel Sutiono, Julia Kurz, Thierry Lonhienne, Jeffrey R. Harmer, Paul V. Bernhardt, Volker Sieber, Luke Guddat, and Gerhard Schenk*

DNA sequences of the enzymes used in this study (the His-tag-encoding sequences are shown in red)

_Sa_DHAD

CATGGGCAGCAGCCATCATCATCATCATCACAGCAGCGGCCTGGTGCCGCGCGGCAGCCATATGCGCAGCGA
TATGATTAAAAAAGGTGATCATCAGGCCCCGGCACGTAGCCTGCTGCATGCAACCGGTGCCCTGAAAAGTCC
GACCGATATGAATAAACCGTTTGTTGCAATTTGTAACAGCTATATTGACATTGTGCCGGGCCATGTGCATCTGC
GTGAACTGGCCGATATTGCAAAAGAAGCCATTCGCGAAGCAGGTGCCATTCCGTTTGAATTTAATACCATTGG
CGTGGATGATGGTATTGCAATGGGTCATATTGGTATGCGCTATAGCCTGCCGAGCCGCGAAATTATTGCCGAT
GCAGCCGAAACCGTGATTAATGCCCATTGGTTTGATGGTGTGTTTTATATTCCGAATTGTGATAAAATCACCCC
GGGCATGATTCTGGCAGCCATGCGCACCAATGTGCCGGCAATTTTTTGCAGTGGTGGCCCGATGAAAGCAGG
CCTGAGTGCCCACGGTAAAGCACTGACCCTGAGCAGCATGTTTGAAGCAGTTGGTGCCTTTAAAGAAGGTAG
TATTAGCAAAGAAGAGTTTCTGGATATGGAACAGAATGCCTGTCCGACCTGCGGTAGTTGCGCCGGCATGTTT
ACCGCCAATAGCATGAATTGTCTGATGGAAGTGCTGGGTCTGGCCCTGCCGTATAATGGTACCGCACTGGCCG
TGAGCGATCAGCGCCGTGAAATGATTCGCCAGGCCGCATTTAAACTGGTTGAAAATATTAAAAACGACCTGAA
ACCGCGTGATATTGTGACCCGCGAAGCAATTGATGATGCATTTGCACTGGATATGGCAATGGGTGGCAGTAC
CAATACCGTGCTGCATACCCTGGCCATTGCAAATGAAGCCGGCATTGATTATGATCTGGAACGCATTAATGCA
ATTGCAAAACGTACCCCGTATCTGAGCAAAATTGCACCGAGTAGTAGCTATAGCATGCATGATGTGCATGAAG
CAGGCGGCGTTCCGGCAATTATTAATGAACTGATGAAAAAAGACGGCACCCTGCATCCGGATCGTATTACCGT
TACCGGTAAAACCCTGCGTGAAAATAATGAAGGCAAAGAAATTAAGAACTTCGATGTGATTCACCCGCTGGAT
GCCCCGTATGATGCCCAGGGCGGCCTGAGCATTCTGTTTGGTAATATTGCACCGAAAGGTGCAGTTATTAAAG
TGGGTGGTGTGGATCCGAGCATTAAAACCTTTACCGGCAAAGCAATTTGTTTTAATAGTCATGATGAGGCCGT
GGAAGCAATTGACAATCGTACCGTGCGCGCCGGTCATGTTGTGGTTATTCGTTATGAAGGTCCGAAAGGTGG
CCCGGGTATGCCGGAAATGCTGGCACCGACCAGTAGCATTGTGGGTCGTGGCCTGGGTAAAGATGTTGCACT
GATTACCGATGGTCGCTTTAGCGGTGCCACCCGCGGTATTGCAGTGGGTCATATTAGTCCGGAAGCCGCCAGT
GGCGGTCCGATTGCCCTGATTGAAGATGGTGATGAAATTACCATTGATCTGACCAATCGTACCCTGAATGTTA
ATCAGCCGGAAGATGTTCTGGCCCGTCGTCGCGAAAGCCTGACCCCGTTTAAAGCCAAAGTGAAAACCGGCT
ATCTGGCACGTTATACCGCCCTGGTTACCAGTGCCAATACCGGCGGTGTGATGCAGGTGCCGGAAAATCTGAT
TTA

_Cj_DHAD

CATGGGCAGCAGCCATCATCATCATCATCACAGCAGCGGCCTGGTGCCGCGCGGCAGCCATATGCGCAGTGA
TGCAATTAAAAAAGGTCATCTGCGTGCCCCGAATCGCAGCCTGCTGCGCGCATGTGGCCTGAAAGATGAAGA
TTTTGATAAACCGTTTATCGGTGTGGCAAATAGCTATATTGATATTATTCCGGGCCATTATTTCCTGAATGATTA
TGCCAAAATCATCAAAGATGAGATTCGTAAAAACGGTTGCGTTCCGTTTGAATTTAATACCATTGGCGTTGAT
GATGGCATTGCAATGGGTCATGAAGGCATGCTGTATAGTCTGCCGAGTCGTGAACTGATTGCCAATAGTATTG
AAACCGTTATGAATGCCCATCAGCTGGATGCCCTGATTTGCATTCCGAATTGTGATAAAATTACCCCGGGTATG
CTGATGGGCGCCCTGCGCGTGAATGTGCCGACCATTTTTGTGAGCGGTGGCCCGATGGCAAGCGGCGTGACC
AAAAAAGGCGAAAAAATTAGTCTGAGTAGCGTTTTTGAAGCAGTTGGTGCATACGAAAGCAAAAAAATTAGT
GAAGAAGAGTTCAAGGACATTGAATGCAGCGCCTGCCCGAGCGGTGGTAGCTGCAGTGGCATGTTTACCGCA
AATAGCATGAATACCCTGTGTGAAGCAATGGGTATTGCACTGGAAGGTAATGGCACCATTCTGGCACTGAGC
AAAGAACGCGAAGAACTGCTGCGCAAAGCCGCCCGTCGTATTTGTGAAATTGCCCTGGATGAACGTTTTAAAA
TTCGCAATATTATCACCCAGAAGGCAGTTCGCAATGCAATGGTTGTTGATATGGCAATGGGTGGTAGCAGCAA
TACCGTTCTGCACATGCTGGCAATTAGCCGTGAAGCAGGTGTGGCCCTGGATATTAAAGATCTGAATTTTATT
AGCAGCAAGGTGGCACATATTGCAAAAATTGCCCCGAGTCTGAATAGCGTTTATATGGATGATATTCATAAGG
CCGGTGGCGTTAGTGCAGTTATGGCAGAAATTAGCAGTCGTCAGGGCCATATTCTGGAACTGGATGCACTGA
CCATTACCGGCGAAAGCCTGAAAGAACGTCTGAAAAATGCAAAAATTAAGGATGAAAACATCATCCGCAAAG
TGGATAATGCCTATAGTAAAGTGGGTGGTCTGGCCATTCTGTTTGGCAATCTGGCCAAACAGGGCTGCGTGAT
TAAAACCGCCGGTATTATTGGCGAACGCAAATTTAAAGGTAAAGCAGTGTGCTTTAACAGTCAGGATGAAGC
CATTAAAGGCATTATTAAAGGCAAAGTGCAGAAAGGCAATGTTTGTGTTATTCGCTATGAAGGCCCGAAAGG
CGGCCCGGGCATGCAGGAAATGCTGAGCCCGACCAGTCTGCTGATGGGTATGGGTCTGGGTGCAGATGTGG

CCCTGATTACCGATGGCCGTTTTAGCGGCGCAACCCGTGGTCTGAGTGTGGGCCATATTAGCCCGGAAGCAG
CCGAAGGTGGTCTGATTGGCCTGCTGAAAGATGGCGATGAAATTGAAATTGATGTTGATGCCTATACCATTCA
TGCAAATGTTAGTGAAGAAGAAATCGCCAAACGCAAAAAAGAATTTGCACTGCCGCAGAAAGAAGTTAGTAG
TCGCTGGCTGCGCATGTATCAGAAACTGGTTAGTAATGCCAGTAAAGGCGCAGTTCTGGATATGGAATAA

Protein sequences of enzymes used in this study (the His-tag leader sequences are not included)

***Sa*DHAD**

MRSDMIKKGDHQAPARSLLHATGALKSPTDMNKPFVAICNSYIDIVPGHVHLRELADIAKEAIREAGAIPFEFNTIG
VDDGIAMGHIGMRYSLPSREIIADAAETVINAHWFDGVFYIPNCDKITPGMILAAMRTNVPAIFCSGGPMKAGLS
AHGKALTLSSMFEAVGAFKEGSISKEEFLDMEQNACPTCGSCAGMFTANSMNCLMEVLGLALPYNGTALAVSDQ
RREMIRQAAFKLVENIKNDLKPRDIVTREAIDDAFALDMAMGGSTNTVLHTLAIANEAGIDYDLERINAIAKRTPYLS
KIAPSSSYSMHDVHEAGGVPAIINELMKKDGTLHPDRITVTGKTLRENNEGKEIKNFDVIHPLDAPYDAQGGLSILF
GNIAPKGAVIKVGGVDPSIKTFTGKAICFNSHDEAVEAIDNRTVRAGHVVVIRYEGPKGGPGMPEMLAPTSSIVGR
GLGKDVALITDGRFSGATRGIAVGHISPEAASGGPIALIEDGDEITIDLTNRTLNVNQPEDVLARRRESLTPFKAKVKT
GYLARYTALVTSANTGGVMQVPENLI

***Cj*DHAD**

MRSDAIKKGHLRAPNRSLLRACGLKDEDFDKPFIGVANSYIDIIPGHYFLNDYAKIIKDEIRKNGCVPFEFNTIGVDDG
IAMGHEGMLYSLPSRELIANSIETVMNAHQLDALICIPNCDKITPGMLMGALRVNVPTIFVSGGPMASGVTKKGEK
ISLSSVFEAVGAYESKKISEEEFKDIECSACPSGGSCSGMFTANSMNTLCEAMGIALEGNGTILALSKEREELLRKAAR
RICEIALDERFKIRNIITQKAVRNAMVVDMAMGGSSNTVLHMLAISREAGVALDIKDLNFISSKVAHIAKIAPSLNSV
YMDDIHKAGGVSAVMAEISSRQGHILELDALTITGESLKERLKNAKIKDENIIRKVDNAYSKVGGLAILFGNLAKQGC
VIKTAGIIGERKFKGKAVCFNSQDEAIKGIIKGKVQKGNVCVIRYEGPKGGPGMQEMLSPTSLLMGMGLGADVALI
TDGRFSGATRGLSVGHISPEAAEGGLIGLLKDGDEIEIDVDAYTIHANVSEEEIAKRKKEFALPQKEVSSRWLRMYQK
LVSNASKGAVLDME

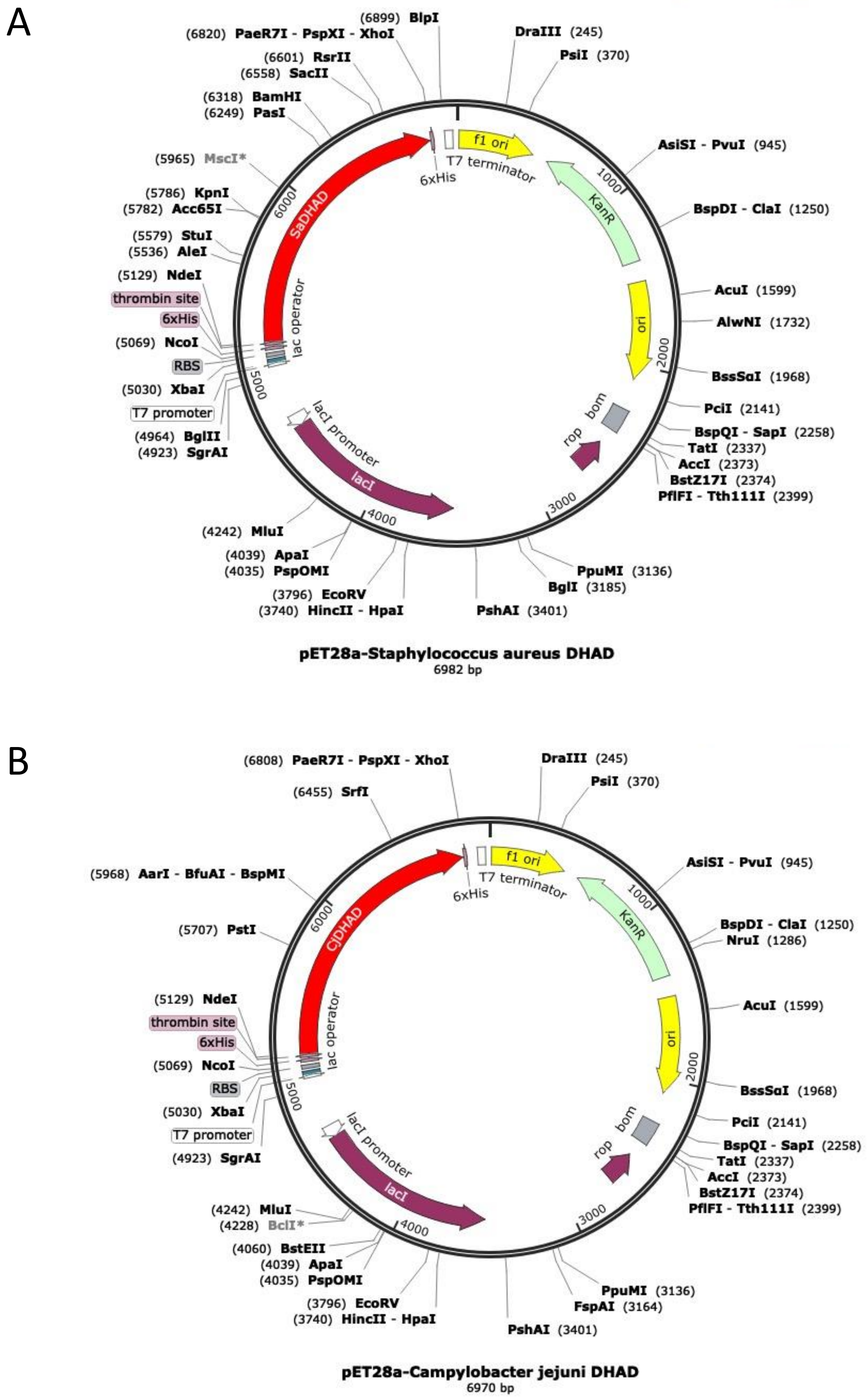

Figure S1. pET28a vector maps used in this study containing the DHAD (ilvD) genes of *S. aureus* (*Sa*DHAD) (A) and *C. jejuni* (*Cj*DHAD) (B). The vectors employ the kanamycin resistance gene for selection.

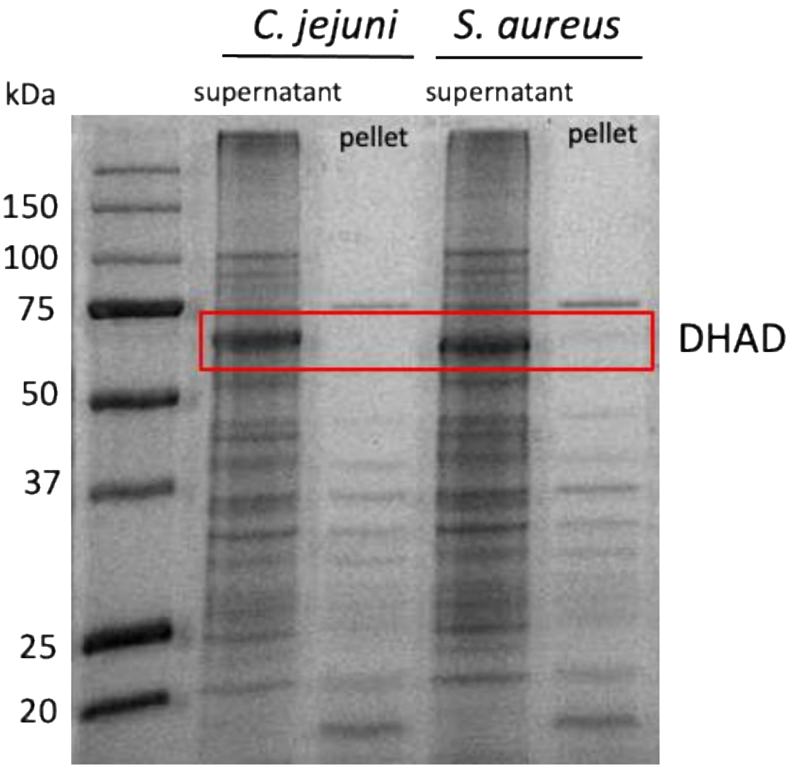

Figure S2. SDS-PAGE of expression of *Cj*DHAD and *Sa*DHAD. The two enzymes can be seen as dark bands between 75 and 50 kDa in the supernatant samples (red box).

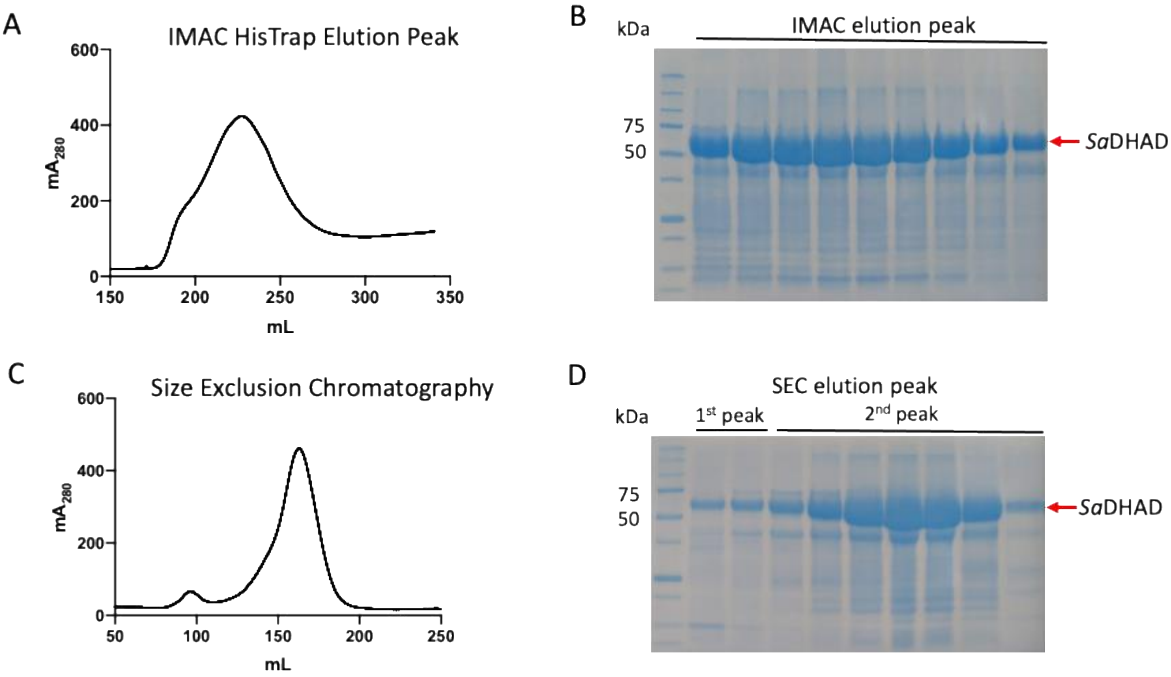

Figure S3. Purification of *Sa*DHAD. A. Chromatogram from the IMAC purification. B. SDS-PAGE with fractions from the IMAC chromatography. C. Chromatogram of the size exclusion purification. D. SDS-PAGE with relevant fractions from the size exclusion purification.

A

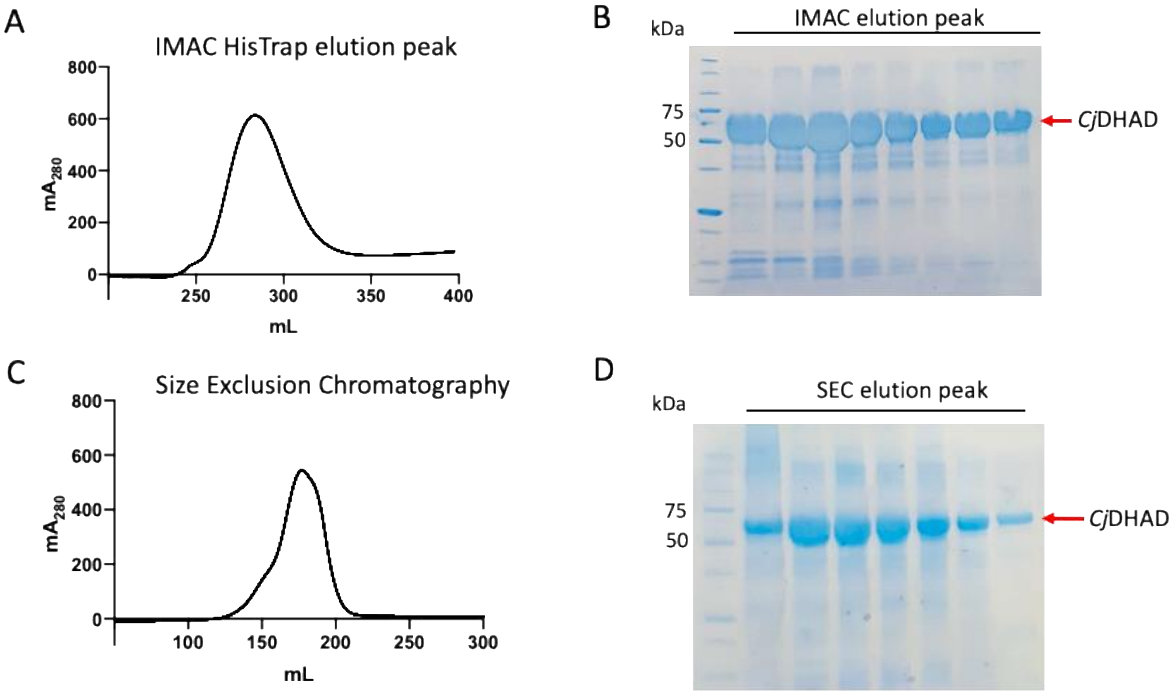

Figure S4. Purification of *Cj*DHAD. A. Chromatogram from the IMAC purification. B. SDS-PAGE with fractions from the IMAC chromatography. C. Chromatogram of the size exclusion purification. D. SDS-PAGE with relevant fractions from the size exclusion purification.

A

Reagents (mM)	Condition 1	Condition 2	Condition 3
Sodium dithionite	20	50	100
2-ME	100	200	400
Ammonium ferrous sulfate	1	10	20

B

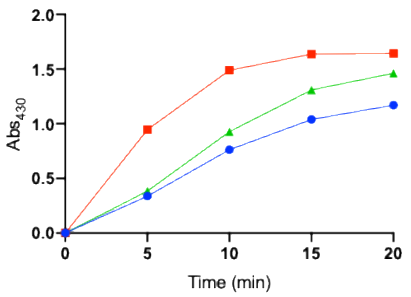

Figure S5. The different conditions tested for the activation of *Cj*DHAD under aerobic conditions. A. Table showing the different concentrations of reducing agents and ammonium ferrous sulfate used. B. Reaction curves corresponding to the three different activation conditions.

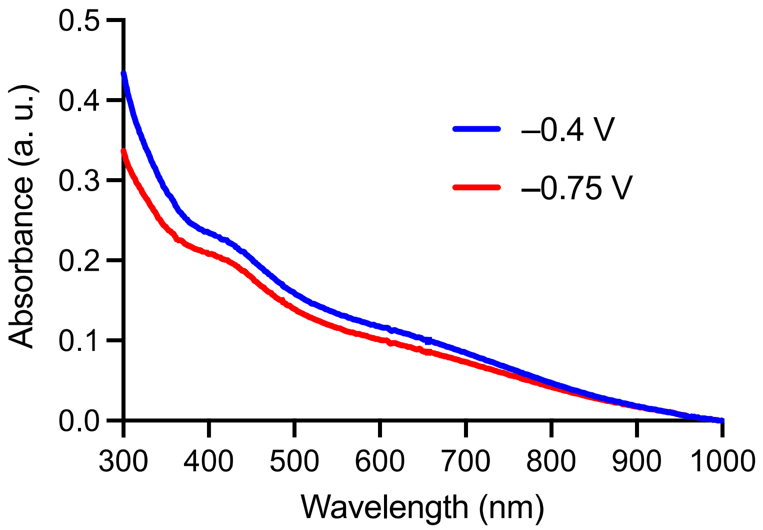

Figure S6. UV-vis spectra observed at potentials –0.4 (blue) and –0.75 V (red) *vs* Ag/AgCl (*i. e.* –0.2 V and –0.55 V *vs* NHE) are not significantly different. The peak at 410 nm characteristic of oxidised [4Fe-4S] cluster is present in both spectra.

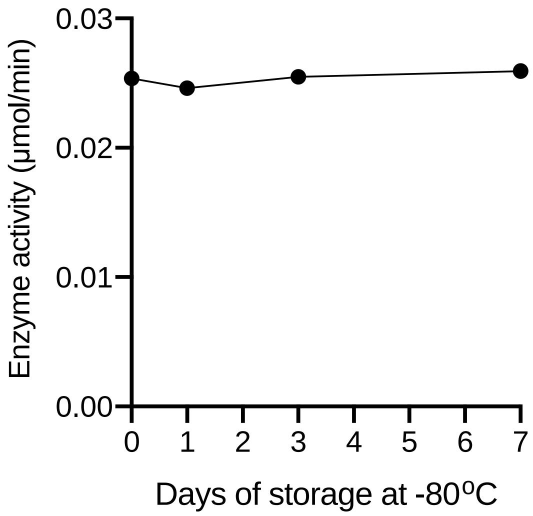

Figure S7. The activity of *Sa*DHAD at days 0, 1, 3 and 7 after aerobic activation during storage at −80 ºC.

Full synthesis of DHIV:

Ethyl 3-methylbut-2-enoate: Synthesised according to a modified procedure by Frei *et al.*[1] 3-methylbut-2-enoic acid (7.4 g, 74 mmol) was dissolved in absolute ethanol (20 mL). Concentrated sulfuric acid (15 drops) was added and the mixture refluxed for 36 h. The reaction mixture was concentrated *in vacuo*, diluted with diethyl ether, and washed with a solution of sat. $NaHCO_3$ (x3) and water (x3). The organic phase was dried with Na_2SO_4 and concentrated *in vacuo* to yield the title compound as a pale-yellow oil (9.3 g, 98%). ^{1}H NMR (300 MHz, $CDCl_3$): δ 5.67 (sept, *J* = 1.3, 1H, CH), 4.14 (q, 7.1, 2H, CH_2), 2.16 (d, *J* = 1.3, 3H, CH_3), 1.88 (d, *J* = 1.3, 3H, CH_3), 1.27 (t, *J* = 7.1, 3H, CH_3). ^{13}C NMR (60 MHz, $CDCl_3$): δ 166.8 (C=O), 156.4 (**C**=CH), 116.1 (CH), 59.4 (CH_2), 27.4 (CH_3), 20.1 (CH_3), 14.3 (CH_2**C**H_3). NMR spectra are consistent with the literature [1]

Ethyl (±)-3,3-dimethyloxirane-2-carboxylate: Ethyl 3-methylbut-2-enoate (5 g, 39 mmol, 1 eq) and 70% *m*-chloroperbenzoic acid (11.5 g, 46.8 mmol, 1.2 eq) were combined in chloroform (25 mL) and refluxed for 17 h. The mixture was allowed to cool, and the white precipitate filtered off. The filtrate was evaporated *in vacuo* then 25% chloroform in hexane was added and the mixture filtered again. The filtrate was concentrated again *in vacuo*, re-dissolved in chloroform, and washed with sat. $NaHCO_3$ solution (x3) and water (x3). A crude colourless oil was obtained (4.24 g), which was purified by column chromatography (10% EtOAc, 0.5% triethylamine in hexane) to obtain the title compound as a colourless oil (2.56 g, 45%). ^{1}H NMR (300 MHz, $CDCl_3$): δ 4.27 (dq, *J* = 7.2 & 15.3, 1H, CH_{2b}), 4.24 (dq, *J* = 7.2 & 15.3, 1H, CH_{2a}), 3.32 (s, 1H, CH), 1.42 (s, 3H, CH_3), 1.38 (s, 3H, CH_3), 1.31 (t, *J* = 7.1, 3H, CH_3). ^{13}C NMR (75 MHz, $CDCl_3$): δ 168.5 (C=O), 61.3 (C-O), 60.2 (CH-O), 59.4 (CH_2), 24.3 (CH_3), 18.2 (CH_3), 14.4 (CH_3). NMR spectra are consistent with the literature [2].

Ethyl (±)-2,3-dihydroxy-3-methylbutanoate: To ethyl 3,3-dimethyloxirane-2-carboxylate (1.37 g, 9.5 mmol) in THF (10 mL) was added 5% aq. HCl (10 mL). The mixture was stirred at rt for 17 h. The THF was evaporated *in vacuo* and adjusted to pH 8 with solid $NaHCO_3$. The product was then extracted with diethyl ether (x3). The organic phase was dried with sodium sulfate and concentrated in vacuo to yield the crude product as a colourless oil (1.54 g) which was purified by column chromatography (25% EtOAc/hexane) to yield the title compound as a colourless oil (840 mg, 56%). ^{1}H NMR (300 MHz, $CDCl_3$): δ 4.32 (dq, *J* = 7.2 & 15.4, 1H, CH_{2b}), 4.28 (dq, *J* = 7.2 & 15.3, 1H, CH_{2a}) 3.95 (d, *J* = 6.7, 1H, OH), 3.15 (d, *J* = 6.7, 1H, CH), 2.60 (s, 1H, OH), 1.33 (t, *J* = 7.1, 3H, CH_3), 1.29 (s, 3H, CH_3), 1.22 (s, 3H, CH_3). ^{13}C NMR (75 MHz, $CDCl_3$): δ 173.2 (C=O), 77.1 (CH-OH), 72.0 (C-OH), 62.1 (CH_3), 25.6 (CH_3), 24.9 (CH_3), 14.2 (CH_3). ^{1}H NMR spectrum is consistent with the literature [3].

(±)-2,3-Dihydroxy-3-methylbutanoic acid: Ethyl 2,3-dihydroxy-3-methylbutanoate (361 mg, 2.2 mmol, 1 eq) and sodium hydroxide (534 mg, 13.3 mmol, 6 eq) were combined in 1:1 THF/H_2O (10 mL) and stirred at rt for 17 h. The reaction mixture was adjusted to pH < 6 with 5% HCl, and then washed with diethyl ether to remove any non-polar impurities. The aqueous phase was then evaporated to dryness *in vacuo*. Absolute ethanol was added, and the mixture heated to reflux for 3 h to ensure the product was completely dissolved. The hot mixture was filtered to remove the insoluble NaCl and the filtrate concentrated *in vacuo* to yield the title compound as a white solid (300 mg, quant.). ^{1}H NMR (300 MHz, DMSO-d_6): δ 6.49 (s, 1H, OH), 4.32 (d, *J* = 3.0, 1H, OH), 3.16 (d, *J* = 2.7, 1H, CH), 1.05 (s, 3H, CH_3), 0.86 (s, 3H, CH_3). ^{13}C NMR (75 MHz, DMSO-d_6): δ 176.0 (C=O), 76.2 (CH-OH), 70.8 (C-OH), 27.8 (CH_3), 23.9 (CH_3). NMR spectra are consistent with the literature [4].

References:

[1] A. Frei, B. Spingler, R. Alberto, *Chem. Eur. J.* **2018**, *24*, 10156-10164.

[2] J. E. Aho, A. Piisola, K. Syam Krishnan, P. M. Pihko, *Eur. J. Org. Chem* **2011**, *2011*, 1682-1694.

[3] T. Kazuhiko, Y. Nobuyuki, T. Rikuhei, K. Aritsune, *Bull. Chem. Soc. Jpn.* **1979**, *52*, 3619-3625.

[4] E. A. Cioffi, K. J. Shaw, W. F. Bailey, C. M. Berg, *Anal Biochem* **1980**, *104*, 485-488.
